# Ebselen Suppresses Breast Cancer Tumorigenesis by Inhibiting YTHDF1-Mediated c-Fos Expression

**DOI:** 10.3390/ijms26199416

**Published:** 2025-09-26

**Authors:** Arathy Vasukutty, Poshan Yugal Bhattarai, Hong Seok Choi

**Affiliations:** Research Institute of Pharmaceutical Sciences, College of Pharmacy, Chosun University, Gwangju 61452, Republic of Korea; v.arathy25@gmail.com (A.V.); poshanb@chosun.ac.kr (P.Y.B.)

**Keywords:** ebselen, breast tumorigenesis, c-Fos proto-oncogene, YTHDF1, m^6^A reader

## Abstract

YTHDF1, an N6-methyladenosine (m^6^A)-binding protein, plays a key role in breast cancer progression, yet its therapeutic targeting remains underexplored. In this study, we investigated the anticancer effects of the novel YTHDF1 inhibitor ebselen in breast cancer cells. Ebselen treatment reduced cell viability in a dose-dependent manner and induced apoptosis, as demonstrated by Annexin V staining, Sub-G1 accumulation, and DNA fragmentation. Consistently, ebselen increased reactive oxygen species (ROS) production and impaired autophagy induction. Mechanistically, ebselen impaired YTHDF1-mediated stabilization and translation of FOS mRNA, leading to decreased c-Fos expression. In addition, ebselen suppressed anchorage-independent growth in vitro and significantly reduced tumor growth in an orthotopic mouse model. These findings highlight YTHDF1 as a promising therapeutic target and support ebselen as a potential small-molecule inhibitor for breast cancer treatment.

## 1. Introduction

Breast cancer is the most common cancer among women worldwide, with an estimated 2.3 million new cases diagnosed globally each year [1]. Although significant progress has been made in breast cancer treatment, challenges such as recurrence and high mortality underscore the urgent need for a definitive cure [2]. Research on breast cancer therapy encompasses a range of strategies, including targeted therapies, immunotherapy, precision medicine, and novel drug combinations, all aimed at improving treatment efficacy and patient outcomes [3,4]. These approaches fundamentally focus on developing drugs that disrupt key cancer hallmarks. As a result, the identification of molecular targets has become a critical force in advancing targeted cancer therapies through innovative technologies and approaches [5].

Advances in breast cancer research have accelerated the development of small-molecule inhibitors that target key molecular pathways essential for tumor growth and survival [6,7,8]. Selenium derivatives exhibit potent antiproliferative activity in various biological assays and enhance the efficacy of chemotherapeutic agents [9]. These effects are mediated through mechanisms such as reactive oxygen species (ROS) generation, modulation of antioxidant defenses, regulation of cell signaling and autophagy, induction of apoptosis, interference with protein kinase pathways, cell cycle arrest, and increasing sensitivity to apoptosis inducers like doxorubicin [10]. Ebselen [N-phenyl-1,2-benzisoselenazol-3(2H)-one] is a synthetic selenium derivative that was initially identified for its glutathione peroxidase-mimicking activity [11]. Its therapeutic potential includes antioxidant, neuroprotective, cardioprotective, antimicrobial, and chemotherapeutic properties, as well as utility in treating diabetes-related disorders and in detoxification [12,13]. In a comparative study of selenium compounds on breast cancer cell lines, ebselen exhibited notable antiproliferative and antimetastatic effects [14]. It has also been shown to inhibit the growth of lung cancer cells through apoptosis induction, cell cycle arrest, and glutathione depletion, highlighting its potential as a cancer therapeutic [13]. However, despite its demonstrated anticancer activity, the specific molecular targets of ebselen responsible for these effects remain poorly defined.

YTH N6-Methyladenosine RNA Binding Protein F1 (YTHDF1), a member of the YTH domain family, is an N6-methyladenosine (m^6^A)-binding protein that regulates mRNA stability and translation through epitranscriptomic regulation. A recent study identified ebselen as a potent, covalent yet reversible inhibitor of YTHDF1, which disrupts m^6^A-dependent mRNA binding by targeting Cys412 near the m^6^A recognition site [15]. Given that YTHDF1 is frequently upregulated in various cancers and associated with poor disease-free survival, it represents a compelling target for anticancer research [16,17]. Inhibitors of YTHDF1 are particularly promising, as they not only suppress tumor cell proliferation but also reprogram the immune microenvironment to promote anti-tumor immunity and enhance immunotherapy efficacy [18,19]. Previously, we demonstrated that YTHDF1 facilitates breast cancer tumorigenesis by stabilizing *AURKA* mRNA [20]. Similarly, several studies have shown that YTHDF1 plays a crucial role in breast cancer tumorigenesis and metastasis [16,21,22]. However, therapeutic targeting of YTHDF1 in breast cancer remains largely unexplored.

c-Fos is a cellular proto-oncogene that dimerizes with c-Jun to form AP-1, a transcription factor complex that activates genes involved in cell proliferation and tumorigenesis [23]. Multiple studies have shown that c-Fos overexpression plays a critical role in breast cancer progression [24,25]. As an immediate-early gene, c-Fos is rapidly induced in response to mitogenic stimuli, and its overexpression in cancer cells is associated with an enhanced tumor microenvironment. However, the role of epitranscriptomic regulation in controlling c-Fos expression remains largely unknown.

In this study, we investigated the anticancer effects of ebselen through inhibition of YTHDF1 in breast cancer cells. We further demonstrated that YTHDF1 promotes c-Fos overexpression—a process that can be disrupted by ebselen. Our findings reveal the therapeutic potential of ebselen in breast cancer and identify the YTHDF1–c-Fos axis as a promising molecular target.

## 2. Results

### 2.1. YTHDF1 Overexpression Predicts Poor Prognosis in Breast Cancer and Can Be Targeted with Ebselen

**Figure 1:**
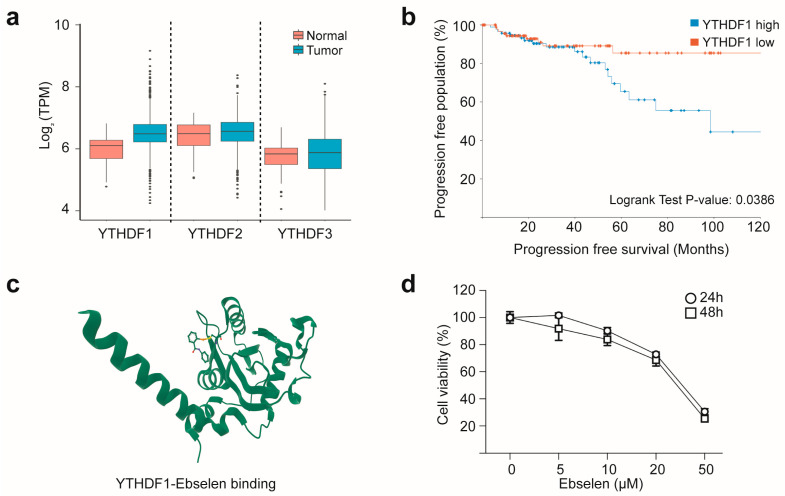
YTHDF1 overexpression in breast cancer represents a viable therapeutic target for ebselen. (**a**) Comparison of RNA expression levels of the YTHDF gene family in breast cancer tissues and matched adjacent normal tissues, based on RNA-seq data from The Cancer Genome Atlas (TCGA). (**b**) Kaplan–Meier plot showing the association between YTHDF1 expression and progression-free survival in the TCGA breast cancer cohort. Patients with breast cancer (*n* = 100 per group) were stratified into high and low YTHDF1 expression groups (data for panels (**a**,**b**) were retrieved from https://portal.gdc.cancer.gov/projects/TCGA-BRCA (accessed on 6 July 2025)). (**c**) Structural model showing ebselen binding to the YTH domain of YTHDF1, specifically near the m^6^A recognition pocket, forming a covalent bond with the Cys412 residue (PDB ID: 7PCU; structure retrieved from https://www.rcsb.org/ (accessed on 8 July 2025)). (**d**) Ebselen induces concentration-dependent cytotoxicity. In MCF7 cells. Cells were treated with the indicated concentrations of ebselen for 24 or 48 h, and cell viability was measured using the MTT assay.

To assess the clinical relevance of m^6^A reader proteins in breast tumorigenesis, we analyzed RNA-seq data from The Cancer Genome Atlas (TCGA) database (https://tcga-data.nci.nih.gov/tcga/ (accessed on 6 July 2025)) by comparing tumor tissues with matched adjacent normal tissues. Among YTHDF family members, YTHDF1 was markedly overexpressed in breast cancer tissues (Figure 1a), consistent with previous reports [16]. Survival analysis using Kaplan–Meier curves (retrieved from https://www.cbioportal.org/ (accessed on 6 July 2025)) revealed that elevated YTHDF1 expression correlated with reduced progression-free survival (Figure 1b), underscoring the clinical significance of YTHDF1. Given the reported affinity of ebselen for YTH domain-containing proteins (Figure 1c), we next examined the anticancer effects of ebselen in breast cancer cells [15]. Cytotoxicity assays using the MCF7 breast cell line demonstrated concentration-dependent effects, with a half maximal inhibitory concentration (IC_50_) of approximately 30 µM, confirming robust sensitivity of MCF7 cells to ebselen (Figure 1d). Taken together, these findings suggest that pharmacological inhibition of YTHDF1 by ebselen exerts anticancer effects, highlighting YTHDF1 as a potential therapeutic target in breast cancer.

### 2.2. Ebselen Inhibits YTHDF1-Induced Elevation of c-Fos Expression

**Figure 2:**
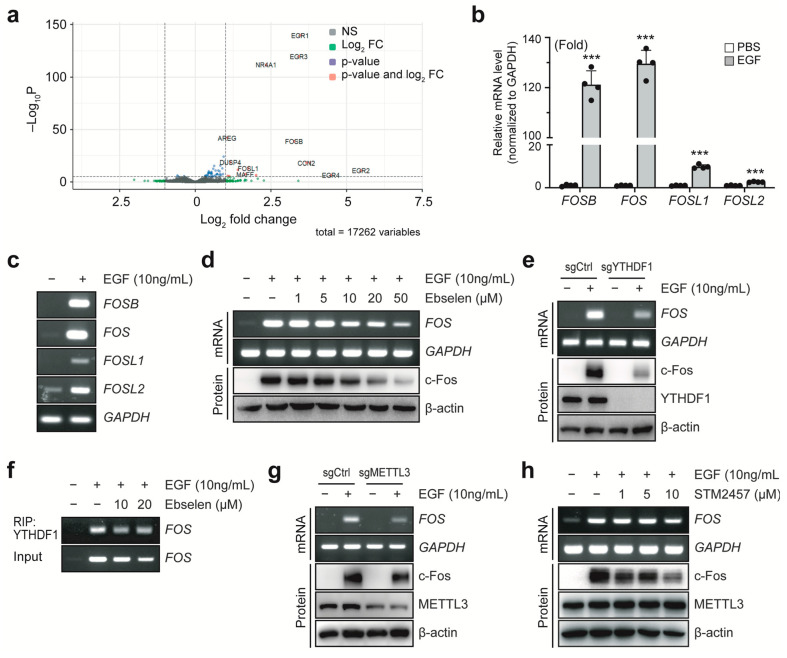
Ebselen regulates YTHDF1-mediated c-Fos expression in MCF7 cells. (**a**) Volcano plot showing mRNAs upregulated by epidermal growth factor (EGF) treatment in a published RNA-seq dataset, with significantly upregulated transcripts highlighted in red. (**b**,**c**) Upregulation of *FOS* gene expression by EGF. MCF7 cells were cultured in serum-free medium for 24 h and treated with EGF for 1 h. Expression of *FOS* family genes was analyzed using real-time (**b**) or end-point (**c**) PCR with gene-specific primers. Data are presented as mean ± SD; *n* = 3. One-way analysis of variance (ANOVA), *** *p* < 0.001. (**d**) Ebselen treatment decreases *FOS* mRNA and protein levels in a concentration-dependent manner. MCF7 cells were treated with varying concentrations of ebselen in serum-free media for 24 h, followed by EGF stimulation. mRNA (**upper panel**) and protein (**lower panel**) expression was analyzed using end-point PCR and western blotting (WB) for the indicated target genes and proteins, respectively. (**e**) Knockout of YTHDF1 reduces *FOS* mRNA and c-Fos protein levels. MCF7 cells transfected with sgCtrl or sgYTHDF1 were serum-starved and treated with EGF. mRNA (**upper panel**) and protein (**lower panel**) expression was analyzed for the indicated target genes and proteins, respectively. (**f**) RNA immunoprecipitation (RIP) analysis using anti-YTHDF1 antibody confirmed the interaction between *FOS* mRNA and YTHDF1 protein. MCF7 cells were serum-starved and treated with varying concentrations of ebselen for 24 h, followed by EGF stimulation. Cell lysates were then subjected to RIP, and the associated RNA was analyzed via PCR. (**g**,**h**) Methyltransferase-like 3 (METTL3) regulates EGF-induced *FOS* expression. MCF7 cells transfected with sgCtrl or sgMETTL3 were serum-starved for 24 h (**g**). or wild-type cells were pre-treated with STM2457 (a METTL3 inhibitor) in serum-free medium (**h**). In both cases, cells were then stimulated with EGF for 1 h, followed by end-point PCR (**upper panel**) and WB (**lower panel**) analysis for the indicated target genes and proteins, respectively.

To investigate the molecular mechanisms underlying the anticancer effects of ebselen, we employed a breast cancer cell model stimulated with epidermal growth factor (EGF). This model recapitulates aspects of the tumor microenvironment, which is typically enriched with growth factors that drive malignant progression. Analysis of a previously published RNA-seq dataset [26] (GSE94408) revealed significant upregulation of several genes associated with cell proliferation, including members of the *FOS* and *EGR* transcription factor families, in EGF-treated MCF7 cells (Figure 2a). The *FOS* family of transcription factors, also known as immediate-early genes, are well-established drivers of breast tumorigenesis [24]. However, the post-transcriptional mechanisms regulating *FOS* mRNA stability remain poorly understood. To address this gap, we focused on *FOS* family regulation in this study. EGF stimulation significantly increased the expression of all *FOS* family members, with *FOS* itself showing the most robust induction (Figure 2b,c). We next examined the effects of ebselen on *FOS* expression. Ebselen treatment reduced both EGF-induced *FOS* mRNA and c-Fos protein levels in a concentration-dependent manner (Figure 2d). To directly assess the role of YTHDF proteins in *FOS* regulation, we individually knocked out YTHDF1–3 in MCF7 cells using CRISPR/Cas9 genome editing. Knockout of YTHDF1, but not YTHDF2 or YTHDF3, resulted in reduced c-Fos expression, indicating that YTHDF1 plays an exclusive role in regulating c-Fos expression (Supplementary Figure S1). Furthermore, EGF-induced upregulation of *FOS* mRNA and protein was markedly attenuated in YTHDF1-deficient cells (Figure 2e), suggesting a functional role for YTHDF1 in stabilizing *FOS* transcripts. RNA immunoprecipitation (RIP) assays for YTHDF1 confirmed that it directly binds to *FOS* mRNA and that this interaction was progressively disrupted by ebselen in a dose-dependent manner, accompanied by a decrease in *FOS* levels (Figure 2f). Given that YTHDF1 binding is dependent on m^6^A modification catalyzed by methyltransferase-like 3 (METTL3), we next examined the role of METTL3 in *FOS* regulation. Both METTL3 knockout and pharmacological inhibition using STM2457 consistently decreased *FOS* mRNA and c-Fos protein levels (Figure 2g,h). Taken together, these findings demonstrate that METTL3 and YTHDF1 cooperatively regulate EGF-induced c-Fos expression.

### 2.3. Ebselen Reduces FOS mRNA Stability

**Figure 3:**
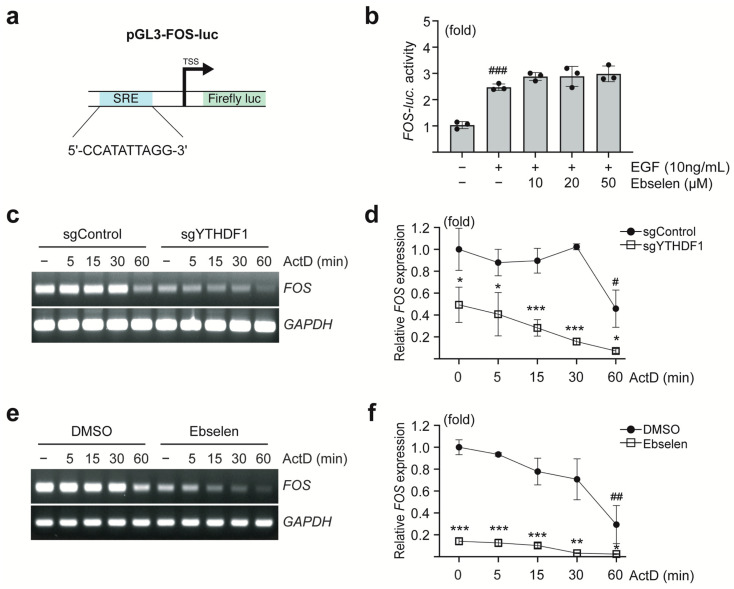
Ebselen reduces *FOS* mRNA stability. (**a**,**b**) Effect of ebselen on *FOS* promoter activity. Schematic representation of the pGL5-FOS reporter construct containing the serum response element (indicated as SRE) consensus sequence in the promoter region of the luciferase reporter gene (**a**). MCF7 cells were transfected with the pGL5-FOS-luc reporter plasmid, followed by ebselen treatment and EGF stimulation (**b**). Firefly luciferase activity was then measured to assess *FOS* promoter activity. Data are presented as mean ± SD; *n* = 3. One-way ANOVA, ^###^ *p* < 0.001. (**c**,**d**) Knockout of YTHDF1 reduces *FOS* mRNA stability. MCF7 cells transfected with sgControl or sgYTHDF1 were treated with actinomycin D (indicated as ActD) for the indicated time points. *FOS* mRNA expression was analyzed using end-point PCR (**c**). Band intensities were quantified via densitometry and normalized to that of *GAPDH* (**d**). Data are presented as mean ± SD; *n* = 3. Student’s *t*-test, * *p* < 0.05, *** *p* < 0.001 (sgControl versus sgYTHDF1 at corresponding time points), ^#^ *p* < 0.05 (sgControl, 0 min versus 60 min) (**d**). (**e**,**f**) Ebselen treatment reduces *FOS* mRNA stability. MCF7 cells were pretreated with ebselen (50 µM) for 24 h, followed by actinomycin treatment at the indicated time points. *FOS* mRNA expression was analyzed using end-point PCR (**e**), and band intensities were quantified via densitometry and normalized to that of *GAPDH* (**f**). Data are presented as mean ± SD; *n* = 3. Student’s *t*-test, * *p* < 0.05, ** *p* < 0.01, *** *p* < 0.001 (DMSO versus ebselen at corresponding time points), ^##^ *p* < 0.01 (DMSO, 0 min versus 60 min).

To investigate the mechanism by which ebselen regulates *FOS* expression, we examined whether it affects the transcriptional activation of the *FOS* gene. A luciferase reporter construct containing the mouse c-*Fos* promoter region, including serum response elements, was used to assess promoter activity (Figure 3a). Ebselen treatment did not attenuate EGF-induced luciferase activity, suggesting that ebselen does not interfere with *FOS* promoter activation (Figure 3b).

In contrast, analysis of *FOS* mRNA stability following actinomycin D treatment revealed that the half-life (t_1/2_) of *FOS* mRNA was significantly reduced in MCF7 cells lacking YTHDF1 (t_1/2_: 58 min) compared to that in control cells (t_1/2_: 19 min) (Figure 3c,d). Similarly, pretreatment of MCF7 cells with ebselen significantly shortened the *FOS* mRNA half-life (t_1/2_: 21 min) compared to that in DMSO-treated cells (t_1/2_: 45 min) (Figure 3e,f). Collectively, these findings indicate that ebselen downregulates *FOS* mRNA levels by decreasing its stability, a process mediated by the inhibition of YTHDF1.

### 2.4. Ebselen Reduces Translation Efficiency of FOS

**Figure 4:**
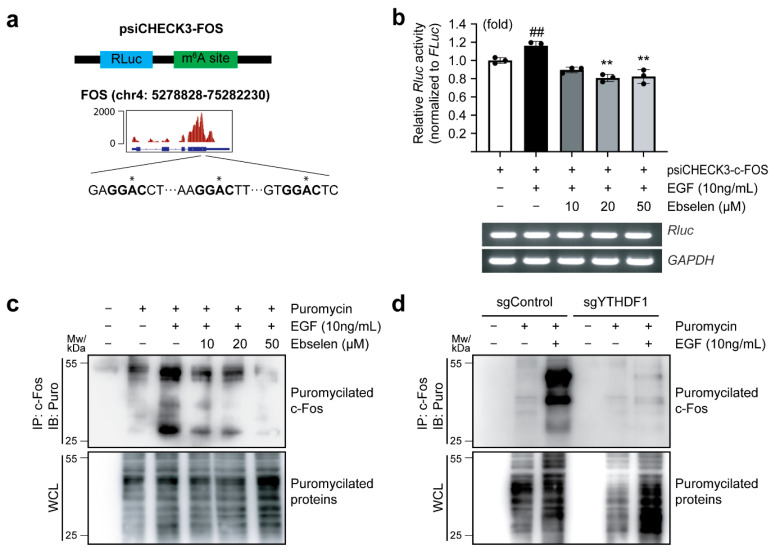
Regulation of *FOS* translation efficiency by ebselen. (**a**) Schematic representation of the psiCHECK3-FO translation reporter construct (upper) and Integrated Genomics Viewer plot showing m^6^A peaks around *FOS* mRNA in MCF7 cells (data from GSM5368774, retrieved from https://www.ncbi.nlm.nih.gov/geo/ (accessed on 2 May 2025)). The sequence of the m^6^A methylation site within the *FOS* 3′ untranslated region is highlighted. The asterisk symbol (*) indicates the methylated adenosine. (**b**) Renilla luciferase (*RLuc*) activity was measured in cell lysates from MCF7 cells transfected with the psiCHECK3-FOS construct and treated with ebselen. *RLuc* activity was normalized to Firefly luciferase activity (*FLuc*) (**upper panel**), and the corresponding mRNA expression in the lysates was analyzed using end-point PCR for the indicated target genes (**lower panel**). Data are presented as mean ± SD; *n* = 3. One-way ANOVA, **^, ##^ *p* < 0.01. (**c**,**d**) Ebselen reduces c-Fos translation efficiency. MCF7 cells were cultured in serum-free medium with indicated concentrations of ebselen for 24 h, followed by puromycin pretreatment for 1 h and EGF treatment for another 1 h. Whole cell lysates (WCLs) were analyzed via immunoprecipitation/immunoblotting (IP/IB) using the indicated antibodies (**c**). MCF7 cells transfected with sgControl or sgYTHDF1 were cultured in serum-free medium for 24 h, followed by pretreatment with puromycin for 1 h and EGF stimulation for another 1 h. WCLs were analyzed via IP/IB using the indicated antibodies (**d**).

YTHDF1 is known to regulate both mRNA stability and translation efficiency [27]. To determine whether YTHDF1 inhibition by ebselen regulates *FOS* mRNA translation efficiency, we first analyzed the published m^6^A-IP-Seq data (GSM5368774) [28] to identify m^6^A modification sites within the 3′ untranslated region (UTR) of FOS mRNA. These identified m^6^A sites were cloned into the 3′-UTR of the Renilla luciferase (*RLuc*) gene in the psiCHECK-3 reporter vector (Figure 4a). Luciferase assays with MCF7 lysates revealed EGF stimulation enhanced RLuc activity when fused to *FOS* m^6^A-containing 3′-UTR, whereas ebselen treatment significantly suppressed this effect (Figure 4b, upper panel). Notably, mRNA levels of ectopically expressed *RLuc* remained unchanged, indicating that ebselen affected translation efficiency rather than mRNA abundance (Figure 4b, lower panel). A puromycin-based labeling assay revealed a dose-dependent suppression of nascent c-Fos synthesis following EGF treatment (Figure 4c). This approach relies on puromycin incorporation into elongating polypeptides, allowing immunodetection of newly synthesized proteins as a direct measure of translational activity. An increased puromycin smear indicates enhanced protein synthesis. To validate the role of YTHDF1, we assessed nascent c-Fos levels in YTHDF1-knockout cells. Knockout of YTHDF1 significantly impaired translation of nascent c-Fos, as evidenced by decreased puromycylation of c-Fos upon EGF treatment (Figure 4d). Notably, global protein synthesis remained largely unaffected by ebselen treatment or YTHDF1 knockout. Taken together, these findings indicate that ebselen selectively impairs the translational efficiency of *FOS* mRNA by inhibiting YTHDF1 expression.

### 2.5. Ebselen Induces Apoptotic Cell Death

**Figure 5:**
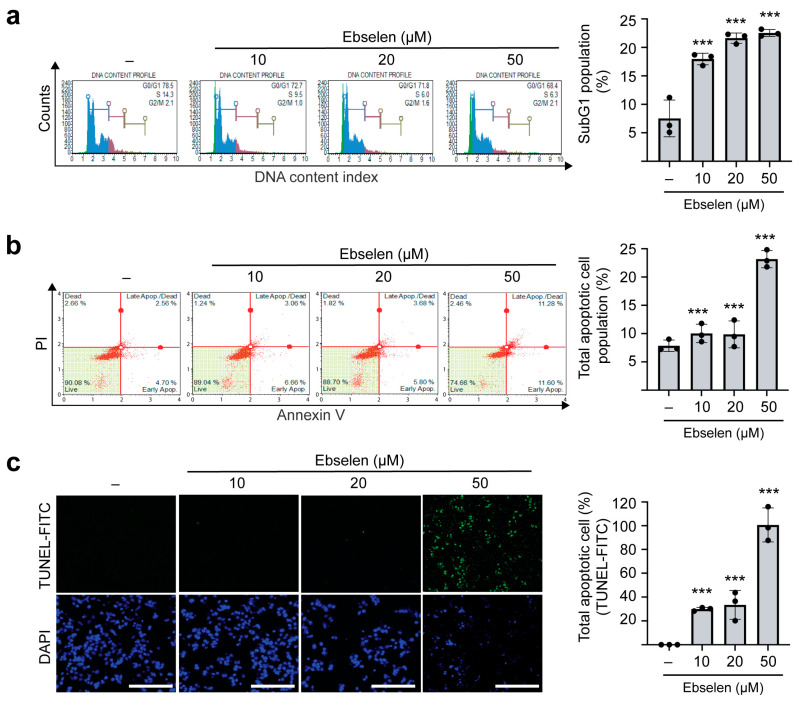
Ebselen induces apoptotic cell death in MCF7 cells. (**a**) Ebselen treatment in MCF7 cells increases the population of sub-G1 cells. Histogram showing cell cycle distribution following ebselen treatment analyzed using the Muse Cell Cycle Analyzer (**left**) and percentage of sub-G1 cell populations across treatments (**right**). Data are presented as mean ± SD; *n* = 3. One-way ANOVA, *** *p* < 0.001. (**b**) Ebselen treatment increases the total number of apoptotic MCF7 cells. Scatter plot of Annexin-V/propidium iodide (PI)-stained MF7 cells following ebselen treatment (**left**) and percentage of apoptotic cells across treatments (**right**). Data are presented as mean ± SD; *n* = 3. One-way ANOVA, *** *p* < 0.001. (**c**) Ebselen treatment enhances DNA fragmentation in MCF7 cells. Cells were stained with terminal deoxynucleotidyl transferase dUTP nick end labeling reagent (green) to detect fragmented DNA and with DAPI (blue) to label nuclei (**left**); scale bar, 300 µM. Quantification of DNA fragmentation (**right**). Data are presented as mean ± SD; *n* = 3. One-way ANOVA, *** *p* < 0.001.

To assess the effects of ebselen on cell cycle progression and apoptosis, MCF7 cells were treated with increasing concentrations of the compound for 48 h. Flow cytometric analysis revealed concentration-dependent accumulation of cells in the sub-G1 phase of the cell cycle (Figure 5a), indicative of apoptotic cell death. Consistently, Annexin V and propidium iodide (PI) staining showed a significant increase in apoptotic cell populations following ebselen treatment (Figure 5b). This was further confirmed through terminal deoxynucleotidyl transferase dUTP nick end labeling (TUNEL) staining, which showed dose-dependent DNA fragmentation characteristic of apoptosis (Figure 5c). Reactive oxygen species (ROS) and autophagy play important roles in apoptotic cell death. Since selenium derivatives including ebselen are well known to induce ROS stress and disrupt autophagic flux [29,30,31], we investigated whether ebselen regulates these processes. Our data showed that ebselen treatment increased ROS generation, as evidenced by enhanced 2′,7′-dichlorodihydrofluorescein diacetate (DCFH-DA) fluorescence staining (Supplementary Figure S2a,b). In addition, ebselen treatment reduced the expression of Beclin-1 and LC3B, key proteins involved in the induction of autophagy, thereby promoting apoptotic cell death (Supplementary Figure S2c). Collectively, these results indicate that ebselen induces dose-dependent cytotoxicity in MCF7 cells primarily by activating apoptotic cell death via ROS generation and inhibition of autophagy.

### 2.6. Ebselen Inhibits Colony Formation and In Vivo Tumorigenesis by Reducing c-Fos Expression

**Figure 6:**
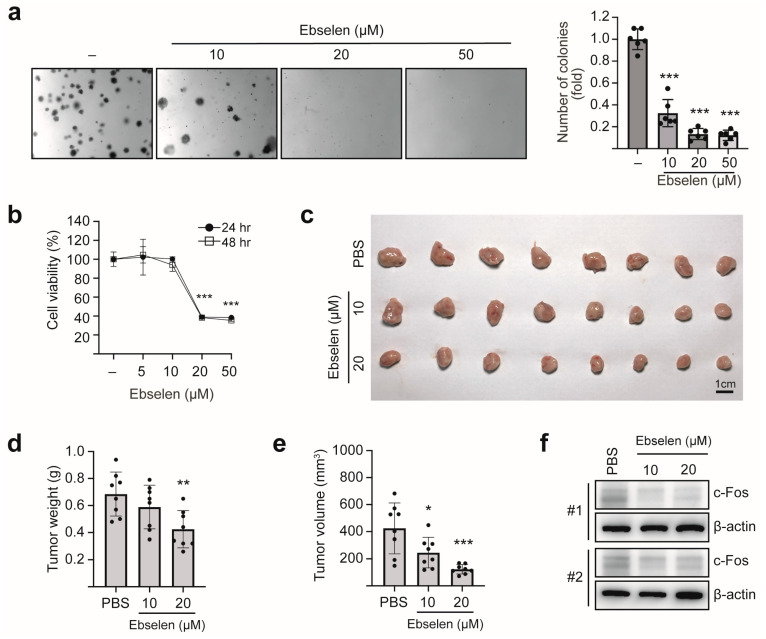
Ebselen suppresses 4T1 breast tumor growth in vivo. (**a**) Ebselen treatment reduces colony formation in MCF7 cells. Representative images show colonies formed by MCF7 cells after treatment with varying concentrations of ebselen (**left**) and quantification of colony formation (**right**). Data are presented as mean ± SD; *n* = 3. One-way ANOVA, *** *p* < 0.001. (**b**) Cytotoxicity of ebselen in 4T1 cells. 4T1 cells were seeded in 96-well plates and treated with different concentrations of ebselen for 24 or 48 h. After treatment, MTT assays were performed to evaluate cell viability. Data are presented as mean ± SD; *n* = 3. One-way ANOVA, *** *p* < 0.001. (**c**–**e**) Inhibition of in vivo tumorigenesis by ebselen. Tumors formed by 4T1 cells pretreated with ebselen in BALB/c mice (**c**), and measurements of tumor weight (**d**) and volume (**e**). Data are presented as mean ± SD; *n* = 8. One-way ANOVA, * *p* < 0.05, ** *p* < 0.01, *** *p* < 0.001. (**f**) Analysis of protein expression in tumor samples via WB for the indicated target proteins.

To evaluate the antitumor potential of ebselen, we first assessed its effects on anchorage-independent growth using soft agar assays in MCF7 cells. Ebselen treatment significantly reduced colony formation (Figure 6a). To validate these findings in vivo, we established an orthotopic breast tumor model using 4T1 cells, a highly aggressive murine breast carcinoma cell line. Ebselen exhibited dose-dependent cytotoxicity against 4T1 cells, as determined by the MTT assay (Figure 6b). For in vivo tumorigenesis analysis, 4T1 cells pretreated with either phosphate-buffered saline (PBS) or ebselen were orthotopically implanted into the mammary fat pads of BALB/c mice. Ebselen treatment significantly reduced both tumor volume and weight in BALB/c mice (Figure 6c–e). Immunoblotting of tumor lysates revealed decreased c-Fos expression following ebselen treatment (Figure 6f). Collectively, these findings demonstrate that ebselen suppresses in vivo breast tumorigenesis by inhibiting c-Fos expression.

## 3. Discussion

M^6^A modification plays a critical role in tumorigenesis, primarily by modulating RNA stability and, to a lesser extent, splicing and subcellular localization. These processes collectively influence the expression and activity of oncogenes and tumor suppressor proteins [32]. Therefore, significant efforts have focused on developing small-molecule inhibitors targeting m^6^A pathways. Potent inhibitors of m^6^A writer proteins, such as STM2457, have been successfully developed [33]. However, therapeutic targeting of m^6^A reader proteins remains unachieved. The m^6^A reader protein YTHDF1 has recently emerged as a key mediator of breast cancer pathogenesis through its role in m^6^A-dependent epitranscriptomic regulation of oncogenic pathways [21]. Analyses of TCGA datasets and prior clinical studies have consistently associated elevated YTHDF1 expression with accelerated tumor progression and reduced disease-free survival in patients, positioning it as both a prognostic biomarker and therapeutic target [27]. Therefore, current drug discovery efforts are focusing on the development of selective small-molecule inhibitors capable of reversibly disrupting YTHDF1′s RNA-binding activity—an approach that may transiently modulate its oncogenic functions while minimizing off-target effects. This study demonstrates the anticancer effects of the selenium derivative, ebselen, in breast cancer through inhibition of YTHDF1.

Selenium-based small-molecule inhibitors have recently garnered considerable interest owing to their potent anticancer properties [9,34]. Ebselen, in particular, has demonstrated notable efficacy in preclinical models of small cell lung carcinoma. Ebselen contains a selenium atom within its ring structure that reacts with thiol (-SH) groups in enzyme active sites, forming covalent selenosulfide (Se–S) bonds, thereby inactivating the target protein. For such inhibition to occur, ebselen must dock into the active site in a favorable orientation. A recent high-throughput screening of organoselenide compounds identified ebselen as a binder of the YTH domain of YTHDF1, where it covalently modifies the cysteine residue Cys412, thereby impairing the protein’s ability to recognize m^6^A-modified RNA [15]. Orthogonal assays confirmed that ebselen similarly targets all three YTHDF paralogs, disrupting their interaction with m^6^A-decorated mRNA. We previously demonstrated that depletion of YTHDF1 suppresses breast tumorigenesis [20]. In this study, we investigated the pharmacological inhibition of YTHDF1 using ebselen in MCF7 breast cancer cells. Cell cytotoxicity assays revealed a significant reduction in cell viability, with an IC_50_ of approximately 30 µM, consistent with a previous report [14].

While c-Fos is recognized as a critical early-response oncogene that modulates multiple tumorigenic pathways [35], the role of epitranscriptomic regulation in controlling c-Fos expression in cancer cells remains unclear. Notably, *FOS* mRNA is inherently unstable, allowing for transient signaling following gene activation. This instability is often exacerbated by miRNA-mediated degradation through binding to the 3′-UTR. YTHDF1 is known to influence mRNA stability by binding to 3′-UTR regions [36], although its direct role in regulating *FOS* mRNA has not been established. In contrast, METTL3 has been reported to upregulate *FOS* mRNA expression in mouse neurons [37]. In this study, we demonstrated that YTHDF1 directly binds to *FOS* mRNA and enhances its stability. Furthermore, we show that METTL3 positively regulates both *FOS* mRNA and c-Fos protein levels, as confirmed through experiments using METTL3 knockout cells and chemical inhibition with STM2457. Importantly, ebselen treatment reduced the elevated *FOS* mRNA stability mediated by METTL3 and YTHDF1 by blocking YTHDF1′s recognition of m^6^A-modified transcripts. This suggests a therapeutic mechanism for ebselen in cancers where FOS drives oncogenic signaling, including breast cancer and potentially other human cancers.

The presence of m^6^A modifications within the 3′-UTR is known to enhance mRNA translation efficiency through YTHDF1 binding. This process involves increased ribosome recruitment and subsequent polysome assembly [38]. Given the prominent m^6^A peaks we observed within the 3′-UTR and around the stop codon, we hypothesized that YTHDF1 may bind to these regions to regulate not only mRNA stability but also translation. Indeed, previous studies have shown that YTHDF1 modulates both the stability and translation efficiency of the same mRNA target, such as NOTCH1, to promote hepatocellular carcinoma [39]. Using psiCHECK3-based translation reporter constructs and nascent protein synthesis assays involving puromycin incorporation, we demonstrated that YTHDF1 regulates translation efficiency and mRNA stability in MCF7 cells. Consistently, ebselen treatment inhibited the enhanced translation of *FOS*, consistent with its destabilizing effect on *FOS* mRNA. Moreover, ebselen-mediated suppression of c-Fos expression led to apoptotic cell death and reduced colony formation. These findings were further validated in 4T1 cells, a highly aggressive murine breast cancer cell line, where ebselen treatment significantly decreased cell viability and suppressed in vivo tumor growth in BALB/c mice.

A major finding of our study is that ebselen induces significant apoptosis. However, the mechanisms driving this apoptosis are likely diverse, as processes such as oxidative stress and autophagy can both trigger apoptotic cell death [40]. Selenium derivatives including ebselen are well known to induce ROS stress and disrupt autophagic flux [29,30,31]. However, whether ebselen triggers cell death in breast cancer via these mechanisms is not reported. We found that ebselen increases ROS generation while reducing the expression of key autophagy-related proteins, suggesting that elevated oxidative stress combined with impaired autophagy-mediated survival contributes to ebselen-induced apoptotic death in breast cancer cells.

In this study, we primarily focused on YTHDF1 as a key target of ebselen. However, a major limitation is that ebselen interacts with all YTHDF proteins as well as numerous other proteins, which complicates the evaluation of individual pathway contributions against breast tumorigenesis. Recent advances in multi-omics technologies have been instrumental in understanding the molecular mechanisms of anticancer drugs [41]. Therefore, integrating datasets generated by next-generation sequencing—including RNA-Seq, m^6^A RIP-Seq, and ribosome profiling—together with proteomics following ebselen treatment could provide a clearer picture of the anticancer mechanisms induced by ebselen.

In summary, our findings advance the existing knowledge of m^6^A methylation in cancer in three key aspects. First, we demonstrate a distinct m^6^A/YTHDF1-dependent regulatory mechanism governing *FOS* mRNA stability. Second, we evaluate the feasibility of using ebselen to modulate *FOS* expression level, providing evidence that ebselen can alter post-transcriptional regulation in a manner linked to m^6^A dynamics. Third, we reveal the therapeutic potential of ebselen in breast cancer treatment, supported by in vivo data that suggest ebselen can inhibit tumor growth or progression, potentially through its effects on *FOS* mRNA stability. Collectively, these findings integrate epitranscriptomic regulation with a clinically relevant compound i.e., ebselen, offering new insights into targeted strategies for breast cancer therapy.

## 4. Materials and Methods

### 4.1. Cell Culture and Generation of CRISPR/Cas9-Mediated Knockout Cells

MCF7 and 4T1 cells purchased from the American Type Culture Collection were maintained in Dulbecco’s modified Eagle’s medium supplemented with 10% fetal bovine serum (FBS) at 37 °C in a humidified atmosphere containing 5% CO_2_. The generation of sgMETTL3 and sgYTHDF1 MCF7 cells and cloning of guide RNA to eSpCas9(1.1)-T2A-Puro vector (Watertown, MA, USA; #101039; a gift from Andrea Nemeth) has been described previously [20,42]. The guide RNA sequences are given in Supplementary Table S1.

### 4.2. Antibodies and Reagents

Antibodies against c-Fos (Clone 9F6; 2250; WB 1:1000), METTL3 (Clone E3F2A; 86132; WB 1:1000), Beclin-1 (3738; WB 1:1000), and YTHDF1 (86463S; WB 1:1000) were purchased from Cell Signaling Technology, Inc. (Danvers, MA, USA). Anti-β-actin antibody (A1987; WB 1:10,000) was purchased from Sigma-Aldrich (St. Louis, MO, USA). The anti-puromycin antibody (MABE343, WB 1:5000) was purchased from Merck Millipore (Burlington, MA, USA). LC3B (NB100-2220; WB 1:5000) was purchased from Novus (St. Charles, MO, USA). Lipofectamine 3000 transfection reagent was purchased from Invitrogen (Carlsbad, CA, USA). 2′,7′-dichlorodihydrofluorescein diacetate (DCFH-DA) was purchased from Sigma-Aldrich (Cat # D6883).

### 4.3. Mammalian Expression Vectors

To design the translation reporter construct, the putative m^6^A modification site of *FOS* (chr4:5278828–75282230) was PCR-amplified from MCF7 cDNA and cloned into the 3′-UTR of *RLuc* at the XhoI site of the psiCHECK3 vector (a gift from Anthony Leung; Addgene plasmid #136010). The pGL3-FOS-Luc plasmid was a gift from Ron Prywes (Addgene plasmid #11983).

### 4.4. Cell Viability Assay

A total of 5000 cells were seeded per well into a 96-well plate containing 100 μL of cell suspension and incubated at 37 °C in a humidified atmosphere with 5% CO_2_. After 24 h, cells were treated with varying concentrations of ebselen and incubated for an additional 24–48 h. Following treatment, 10 μL of EZ-Cytox Cell Viability Assay reagent (Daeli Lab Service, Seoul, Republic of Korea) was added to each well and incubated for another 4 h. The absorbance of the resulting purple formazan, which indicates viable cells, was measured at 450 nm using a microplate reader (Molecular Devices, San Jose, CA, USA).

### 4.5. Protein Immunoblotting

For immunoblotting, cells grown in monolayers were harvested, washed with PBS, and lysed using radioimmunoprecipitation assay (RIPA) buffer containing 150 mM NaCl, 50 mM Tris-HCl (pH 7.4), 0.25% sodium deoxycholate, 1 mM EDTA, 1% NP-40, 1 mM NaF, 0.2 mM PMSF, 0.1 mM sodium orthovanadate, and a protease/phosphatase inhibitor cocktail. Protein lysates (10–30 µg) were separated via SDS-PAGE and transferred onto polyvinylidene fluoride membranes. After incubating the membranes with the appropriate primary and secondary antibodies, protein bands were visualized using the SuperSignal West Femto substrate (Thermo Fisher Scientific, Waltham, MA, USA), and the chemiluminescent signal was detected using the Amersham Imager 680 (GE Healthcare, Chicago, IL, USA).

### 4.6. RIP-PCR

MCF7 cells were harvested using trypsin and lysed in RIP buffer (pH 7.5, 2 mM ribonucleoside vanadyl complexes, 0.1% NP-40, 150 mM NaCl, 10 mM Tris-HCl, and 200 U/mL RNasin). Protein G magnetic Dynabeads (Thermo Scientific) were incubated with 1 µg of YTHDF1 antibody for 1 h at 4 °C in RIP buffer. Antibody-conjugated beads were washed twice with RIP buffer and incubated with 500 µg of clarified cell lysate for 4 h at 4 °C. Following five washes with RIP buffer, RNA bound to the beads was purified and eluted using the AccuPrep^®^ Universal RNA Extraction Kit (Bioneer, Daejeon, Republic of Korea). First-strand cDNA was synthesized from 5 µL of eluted RNA using TOPscript™ RT DryMIX dT18 plus (Enzynomics, Daejeon, Republic of Korea). For endpoint PCR, the cDNA was diluted 1:10 in nuclease-free water. Reverse transcription polymerase chain reaction (RT-PCR) was then performed using AccuPower PCR Premix (Bioneer) with the gene-specific primers given in Supplementary Table S1.

### 4.7. Luciferase Reporter Assay

Firefly luciferase activity was quantified from lysates of MCF7 cells transfected with pGL3-FOS-Luc constructs using the Dual-Luciferase Reporter Assay System (Promega, Madison, WI, USA). Renilla luciferase activity was similarly measured in lysates from MCF7 cells transfected with psiCHECK3 constructs using the same assay system. Firefly luciferase activity encoded by the same vector served as an internal control to normalize for transfection efficiency. Total RNA was extracted from the same lysates for downstream analysis.

### 4.8. Puromycin Labeling and Immunoprecipitation

MCF7 cells, either untreated, treated with ebselen, or edited with sgYTHDF1, were incubated with puromycin (1 µg/mL) for 1 h at 37 °C to label newly synthesized proteins. Following treatment, cells were harvested and lysed using NET-NL buffer containing 50 mM Tris-HCl (pH 7.5), 150 mM NaCl, 0.5% NP-40, 1 mM EDTA, 1 mM dithiothreitol, 0.2 mM PMSF, along with a protease (Roche Life Sciences, Indianapolis, IN, USA), and a phosphatase (Thermo Fisher Scientific) inhibitor cocktail. c-Fos was immunoprecipitated from the lysates using an anti-c-Fos antibody, and the immunoprecipitates were analyzed by immunoblotting with an anti-puromycin antibody to detect puromycin-labeled nascent proteins.

### 4.9. Cell Cycle Analysis

After seeding, cells were treated with various concentrations of ebselen for 48 h, washed, and fixed in 70% ethanol. The fixed cells were then incubated with 200 μL of Muse™ Cell Cycle Reagent (Merck Millipore, Burlington, MA, USA) for 30 min at 25 °C in the dark.

### 4.10. Annexin V–PI Staining

Apoptosis was assessed using the Muse™ Annexin V & Dead Cell Kit (Merck Millipore, Billerica, MA, USA). A 100 μL suspension of MCF7 cells pretreated with various concentrations of ebselen was mixed with an equal volume of Muse™ Annexin V & Dead Cell Reagent containing 1% bovine serum albumin (BSA). Following this, 200 μL of Muse™ Cell Cycle Reagent (Merck Millipore) was added, and the samples were incubated in the dark for 30 min. The stained cells were then analyzed using the Muse Cell Analyzer (EMD Millipore Corporation).

### 4.11. TUNEL Assay

To assess apoptotic cell death, 2 × 10^4^ cells were seeded into each well of a 24-well plate and incubated for 24 h. Cells were then treated with varying concentrations of ebselen for 48 h. Following treatment, cells were washed with PBS and fixed in Cytofix/Cytoperm™ reagent at 4 °C for 20 min. The fixed cells were incubated with 50 µL of TUNEL reaction mixture at 37 °C for 1 h in the dark and then washed twice with PBS. DNA fragmentation was visualized using an Axiovert 200 M fluorescence microscope equipped with a FITC filter.

### 4.12. 2′7′-Dichlorodihydrofluorescein Diacetate (DCFH-DA) Staining Assay

2 × 10^4^ MCF7 cells were plated into each well of a 24-well plate and incubated for 24 h. Cells were subsequently treated with given concentration of ebselen for 48 h, followed by incubation with 25 µM 2′,7′-dichlorodihydrofluorescein diacetate (DCFH-DA) for 30 min. As a positive control, cells were pre-treated with 1 mM hydrogen peroxide (H_2_O_2_) prior to DCFH-DA addition. The fluorescence generated by the oxidized form of DCFH-DA was immediately detected using the green channel on an EVOS M5000 microscope (Invitrogen).

### 4.13. Anchorage-Independent Cell Transformation (Soft Agar Assay)

A total of 8000 cells were treated with ebselen in 1 mL of 0.3% Eagle’s basal medium supplemented with 10% FBS. The cultures were incubated at 37 °C in a humidified atmosphere containing 5% CO_2_ for 14 d. Colony formation was assessed using an Axiovert 200 M microscope and analyzed with AxioVision software, v4.6 (Carl Zeiss, Oberkochen, Germany). Six representative images were captured per treatment group. Colony number and size were quantified using ImageJ software, v1.53t (NIH, Bethesda, MD, USA).

### 4.14. Mouse Orthotopic Model

Six-week-old female BALB/c mice were obtained from Orient Bio (Seongnam, Republic of Korea) and housed under controlled environmental conditions with regulated lighting and temperature. The mice were provided with commercial rodent chow (OrientBio Co., Seongnam, Republic of Korea) and water ad libitum. Animals were randomly assigned to three groups, with the number of mice per group specified for each experiment. 4T1 cells treated with PBS, 10 µM ebselen, or 20 µM ebselen (2 × 10^6^ cells in 100 µL) were orthotopically injected into the abdominal mammary fat pads. Tumor development was monitored daily over a 21-d period. Tumor volume was calculated using the formula: volume = 0.5 × (large diameter) × (small diameter)^2^. The study protocol was approved by the Animal Experiments Committee of Chosun University (CIACUC 2025-A0001).

### 4.15. Statistical Analysis

Comparisons between two independent groups were performed using a two-tailed Student’s *t*-test. For comparisons involving multiple groups, one-way analysis of variance was conducted, followed by Tukey’s post hoc test. A *p*-value less than 0.05 was considered statistically significant.

## Data Availability

The data presented in this study are available on request from the corresponding author.

## Supplementary Materials

The following supporting information can be downloaded at: https://www.mdpi.com/article/10.3390/ijms26199416/s1.

## Abbreviations

The following abbreviations are used in this manuscript:

ANOVA	One-way analysis of variance
BSA	Bovine serum albumin
CRISPR/Cas9	Clustered Regularly Interspaced Short Palindromic Repeats/CRISPR-associated protein 9
DCFH-DA	2′,7′-dichlorodihydrofluorescein diacetate
EGF	Epidermal growth factor
FLuc	Firefly luciferase
IC50	Half maximal inhibitory concentration
m^6^A	N6-methyladenosine
METTL3	methyltransferase-like 3
MTT	3-(4,5-dimethylthiazol-2-yl)-2,5-diphenyltetrazolium bromide
PBS	Phosphate-buffered saline
PI	Propidium iodide
RLuc	Renilla luciferase
RIP	RNA immunoprecipitation
RIPA	Radioimmunoprecipitation assay
ROS	Reactive oxygen species
SRE	Serum response element
TCGA	The Cancer Genome Atlas
TUNEL	Terminal deoxynucleotidyl transferase dUTP nick end labeling
UTR	Untranslated region
WB	Western blotting
WCLs	Whole cell lysates
YTHDF1	YTH N6-Methyladenosine RNA Binding Protein F1
