# Supplementary material for: Ebselen Suppresses Breast Cancer Tumorigenesis by Inhibiting YTHDF1-Mediated c-Fos Expression
Source: Int J Mol Sci. 2025 Sep 26;26(19):9416. doi: 10.3390/ijms26199416 (PMC12524890; doi:10.3390/ijms26199416)
Supplement: Supplementary file 1 [file ijms-26-09416-s001.zip › ijms_Supple Figure_R1.pdf]

Supplementary Figure S1

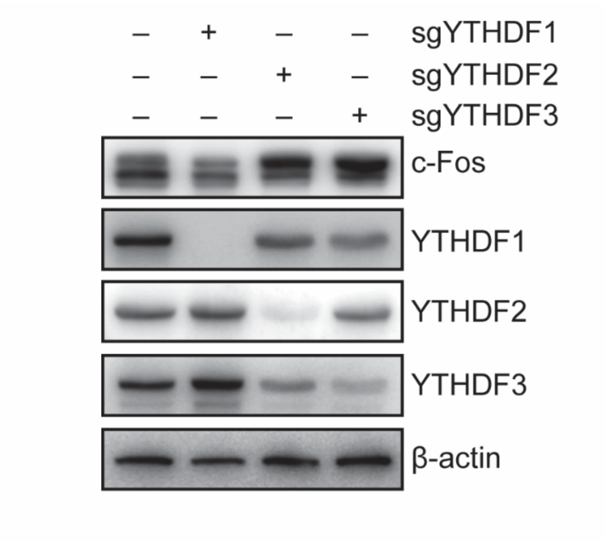

Figure S1. YTHDF1, but not YTHDF2 or YTHDF3, regulates c-Fos expression. MCF7 cells were co-transfected with two sgRNA vectors targeting the indicated gene, while control cells were transfected with an empty vector. Whole-cell lysates were analyzed by western blotting using the specified antibodies.

Supplementary Figure S2

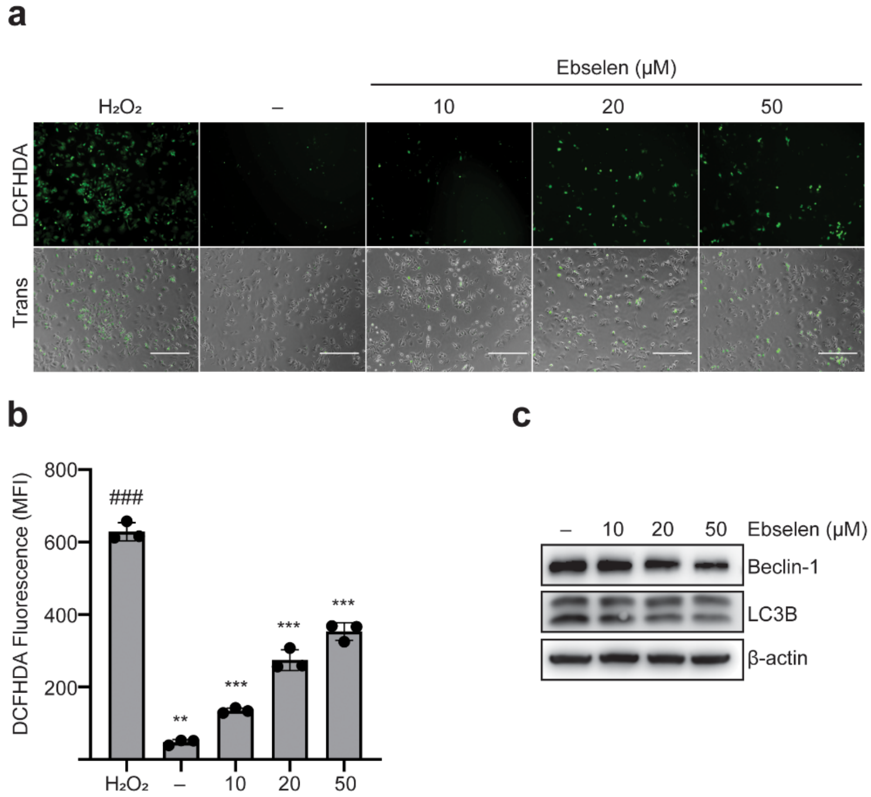

Figure S2. Effect of ebselen treatment in ROS generation and autophagy. **(a-b)** Ebselen promotes ROS production. MCF7 cells were pre-treated with given concentration of ebselen for 48 h, followed by incubation with 25 μM 2′,7′-dichlorodihydrofluorescein diacetate (DCFH-DA) for 30 min. As a positive control, cells were pre-treated with 1 mM hydrogen peroxide (H_2O_2) prior to DCFH-DA addition. Representative fluorescent and corresponding phase contrast images of ROS detection (a), and quantification of mean fluorescence intensity (MFI) using Fiji (b). Data are presented as mean ± SD; n = 3. ANOVA, $^{**}P < 0.01$, $^{***}P < 0.001$, $^{###}$ $P < 0.001$ (compared to control). Scale bar, 300 μM. **(c)** Ebselen inhibits autophagy induction. MCF7 cells were treated with given concentration of ebselen for 48h**.** The whole cell lysate was examined with western blotting using indicated antibodies.
