# Supplementary material for: Ebselen Suppresses Breast Cancer Tumorigenesis by Inhibiting YTHDF1-Mediated c-Fos Expression
Source: Int J Mol Sci. 2025 Sep 26;26(19):9416. doi: 10.3390/ijms26199416 (PMC12524890; doi:10.3390/ijms26199416)
Supplement: Supplementary file 1 [file ijms-26-09416-s001.zip › ijms_Supple table_R1.pdf]

Table S1. List of primers and guide RNAs

	Primer name	Sequence (5' → 3')	Purpose
1	GAPDH (forward)	AACTTTGGTATCGTGGAAGGA	semi-quantitative RT-PCR and real-time PCR
2	GAPDH (reverse)	GAGGCAGGGATGATGTTCT	
3	RLuc (forward)	GTAACGCTGCCTCCAGCTAC	
4	RLuc (reverse)	CCAAGCGGTGAGGTACTTGT	
5	FOS (forward)	CGAGCGAGCTGGTGCATTACAGAG	
6	FOS (reverse)	CGAGTCTCCGGAAGAGGTAAGGAC	
7	FOSB (forward)	GAAGAGGAGAAGCGAAGGGT	
8	FOSB (reverse)	CACCAGCACAAACTCCAGAC	
9	FOSL1 (forward)	GACCTACCCTCAGTACAGCC	
10	FOSL1 (reverse)	TCAGTTCCTTCCTCCGGTTC	
11	FOSL2(forward)	CTCAGGCAGTGCATTCATCC	
12	FOSL2 (reverse)	TCTTGATCACGCCAGGTCTT	
13	YTHDF1 guide RNA1	GGGGTGGGATTCGACGCTGG	CRISPR/Cas9 gene knock out
14	YTHDF1 guide RNA2	GGTGTAGCTGCTCCCATACG	
15	YTHDF2 guide RNA1	GGGTAAGTAGGAATCTGACA	
16	YTHDF2 guide RNA2	GAATACTATAGACCAAGGGA	
17	YTHDF3 guide RNA1	GGTGCTGCACTGCTAACTGG	
18	YTHDF3 guide RNA2	GCAGGCTTCAACCAGAACAA	
